
JOURNAL INFORMATION
==============================
Journal ID: Intereconomics

Intereconomics

EISSN: 1613-964X

ARTICLE INFORMATION
==============================
PMCID: PMC9755757
DOI: 10.1007/s10272-022-1084-z
Article ID: 1084
Article version: 1
Subjects: Forum Introduction

De-Globalisation — A New World Order?

Electronic publication date: 2022 Dec 16
Print publication date: 2022
Volume: 57
Issue: 6
First page: 344
Last page: 344
Copyright: © The Author(s) 2022
License: Open Access: This article is distributed under the terms of the Creative Commons Attribution 4.0 International License (https://creativecommons.org/licenses/by/4.0/).
Open Access funding provided by ZBW — Leibniz Information Centre for Economics.
License URL: https://creativecommons.org/licenses/by/4.0/

issue-copyright-statement: © The Author(s) 2022

==============================

DOI: 10.1007/s10272-022-1084-z

### De-Globalisation – A New World Order?

**After decades of increasing globalisation in trade, capital and information flows, we are currently experiencing a shift due to the COVID-19 pandemic, Russia's war in Ukraine and divisions over the conflict. Disrupted supply chains, US-China trade tensions and the gradual demise of the World Trade Organization appear to be serious threats to globalisation and have altered the geopolitical landscape. Where is globalisation heading? Are we in a temporary phase of slowbalisation or is it the beginning of something different? Is a more multipolar world order developing or one divided between US and Chinese influence? And where does the EU fit in this new scheme? This Forum, featuring contributions by the speakers at the 2022 joint *Intereconomics*/CEPS conference, examines geopolitics and geoeconomics in a changing global order.**

### What Endgame for the Deglobalisation Narrative?

**Simon J. Evenett**, University of St. Gallen; St. Gallen Endowment for Prosperity Through Trade, Switzerland.

### Slowbalisation in the Context of US-China Decoupling

**Alicia Garcia-Herrero**, Bruegel, Brussels, Belgium; Hong Kong University of Science and Technology.

### Reshoring by Decree? The Effects of Decoupling Europe from Global Value Chains

**Alexander Sandkamp**, Kiel University (CAU); Kiel Institute for the World Economy (IfW), Germany.

### What European Union in the "Age of Uncertainty"? Weathering the Geopolitical Storms in a World of Perpetual Crises

**Isabelle Ioannides**, Hellenic Foundation for European & Foreign Policy (ELIAMEP), Athens, Greece.

### The New Globalisation: From Too Big to Fail to Too Intertwined to Decouple

**Simone Urbani Grecchi**, Intesa Sanpaolo, Milan, Italy.

### The End of the End of History: A Political-Economy Perspective

**Ben Zissimos**, University of Exeter, UK.
